# Impacts of socioeconomic and environmental factors on neoplasms incidence rates using machine learning and GIS: a cross-sectional study in Iran

**DOI:** 10.1038/s41598-024-61397-5

**Published:** 2024-05-08

**Authors:** Mohammad Rafiee, Mahsa Jahangiri-rad, Anoushiravan Mohseni-Bandpei, Elham Razmi

**Affiliations:** 1 Air Quality and Climate Change Research Center, Shahid Beheshti University of Medical Sciences, Tehran, Iran; 2 Department of Environmental Health Engineering, School of Public Health and Safety, Shahid Beheshti University of Medical Sciences, Tehran, Iran; 3 Department of Environmental Health Engineering, School of Health, Tehran Medical Sciences, Islamic Azad University, Tehran, Iran; 4 Water Purification Research Center, Islamic Azad University, Tehran, Iran; 5 Department of Environmental Health Engineering, School of Public Health, Iran University of Medical Sciences, Tehran, Iran

**Keywords:** Incidence rate of neoplasms, Multiscale geographical weighted regression, Random forest, Risk factors, Spatiotemporal analysis, Environmental sciences, Risk factors, Engineering

## Abstract

Neoplasm is an umbrella term used to describe either benign or malignant conditions. The correlations between socioeconomic and environmental factors and the occurrence of new-onset of neoplasms have already been demonstrated in a body of research. Nevertheless, few studies have specifically dealt with the nature of relationship, significance of risk factors, and geographic variation of them, particularly in low- and middle-income communities. This study, thus, set out to (1) analyze spatiotemporal variations of the age-adjusted incidence rate (AAIR) of neoplasms in Iran throughout five time periods, (2) investigate relationships between a collection of environmental and socioeconomic indicators and the AAIR of neoplasms all over the country, and (3) evaluate geographical alterations in their relative importance. Our cross-sectional study design was based on county-level data from 2010 to 2020. AAIR of neoplasms data was acquired from the Institute for Health Metrics and Evaluation (IHME). HotSpot analyses and Anselin Local Moran's I indices were deployed to precisely identify AAIR of neoplasms high- and low-risk clusters. Multi-scale geographically weight regression (MGWR) analysis was worked out to evaluate the association between each explanatory variable and the AAIR of neoplasms. Utilizing random forests (RF), we also examined the relationships between environmental (e.g., UV index and PM_2.5_ concentration) and socioeconomic (e.g., Gini coefficient and literacy rate) factors and AAIR of neoplasms. AAIR of neoplasms displayed a significant increasing trend over the study period. According to the MGWR, the only factor that significantly varied spatially and was associated with the AAIR of neoplasms in Iran was the UV index. A good accuracy RF model was confirmed for both training and testing data with correlation coefficients *R*^2^ greater than 0.91 and 0.92, respectively. UV index and Gini coefficient ranked the highest variables in the prediction of AAIR of neoplasms, based on the relative influence of each variable. More research using machine learning approaches taking the advantages of considering all possible determinants is required to assess health strategies outcomes and properly formulate policy planning.

## Introduction

In 2019, neoplasms—a type of aberrant and excessive tissue growth^1^—were projected to have disability adjusted life years (DALYs) of 4,239 per 100,000 people globally^2^. Neoplasia is the term used to describe the process by which tumor forms or is produced^3,4^.

Neoplasms are categorized by ICD-10 (a medical classification list by the World Health Organization [WHO]) into four primary groups, namely benign, malignant, in situ, and neoplasms with unclear or unknown activity^4^. Cancers, usually referred to as malignant neoplasms, are the main focus of current studies as they are one of the driving causes of death and major obstacles to raising life expectancy^5–7^. 482,229 newly diagnosed cancer cases that were registered by the Iranian National Population-Based Cancer Registry between 2014 and 2017 were analyzed in a comprehensive study carried out by Faramarzi and co-authors. To investigate geographical patterns throughout Iran, they used a local Moran I analysis along with a purely spatial scanning model. The results indicated that males accounted for about 53% of all cases. The most frequent malignancy in men was stomach cancer. Iran's northern and northwest was found to have a significant incidence of stomach cancer in both sexes. While, the most prevalent cancer in women, breast cancer, was found to be concentrated in the northern areas of the country^8^.

In the South Iranian province of Kerman, a cross-sectional study was carried out by Montazeri et al.^9^ to examine the spatiotemporal mapping of prostate and breast cancers. high-high clusters of breast cancer were found in the northwest of the province using cluster analysis at the county level; while, high–high clusters related to prostate cancer were found in this region by cluster analysis at the county and district levels.

Globally, the burden of cancer incidence and death is increasing at a rapid pace. This can be attributed to a number of factors, including population increase and aging, as well as shifts in the distribution and frequency of its primary risk factors, many of which are linked to socioeconomic development^10,11^.

The potential impact of environmental exposures on cancer was investigated in a study by Huang et al.^12^. Based on county-level data, they ran a spatial autoregressive model to investigate the relationship between the mortality rates of four cancers in women (breast, cervical, ovarian, and uterine cancer) and the Environmental Quality Index (EQI, which included air, water, land, built environment, and sociodemographic domain). They found a correlation between increased breast, ovarian and uterine cancer mortality and lower environmental quality.

There is unquestionable evidence linking cigarette smoking to the development of several cancers^13^. Higher-energy UV radiation^14^ and air pollution are also two more possible modifiable environmental cancer risk factors^15–17^. Additionally, particulate matter (PM) has been firmly linked to various adverse consequences, including cancer^18,19^. The number of deaths and crude death rate per 100,000 populations in Iran that can be attributed to exposure to ambient PM_2.5_ increased from 36,379 and 47 in 2010 to 41,742 and 49 in 2019, according to the Global Burden of Disease (GBD) study ((https://vizhub.healthdata.org/gbd-compare/).

It is asserted that certain hepatitis strains raise the chance of developing some malignancies. Persistent infections caused by Hepatitis B (HBV) and Hepatitis C (HCV) raise the risk of liver cancer, and the chance of contracting both the hepatitis B and C viruses increases the risk of being afflicted with liver cancer^20^, as well as other types including digestive system cancers^21^. Furthermore, there is an increased risk of bile duct cancer and some non-Hodgkin lymphomas in individuals infected with one or both hepatitis viruses^20,22^. Cervical cancer, the second largest cause of cancer-related mortality among women, is known to be significantly elevated in cases when sexually transmitted diseases (STDs), such as genital warts, Human Immunodeficiency Virus (HIV) and Syphilis, are present^23,24^. The risk of developing other cancer types also grows up following being afflicted with STDs^25^. The primary behavioral drivers of neoplasms, like smoking and environmental exposures, are significantly impacted by socioeconomic conditions at both the individual and local levels^26^. It is crucial to analyze the socioeconomic patterns in cancer mortality and incidence in order to assess the health inequalities associated with cancer between the most and least advantaged social groups and to identify regions or population groups that are most at risk of cancer^27^. In a recent study conducted by Banerjee and Jones (2022), Geographically Weighted Regression (GWR) was utilized to investigate the influence of location on the association between sociodemographic variables and breast cancer. The results revealed that the age-adjusted breast cancer incidence rate per 100,000 were significantly predicted by the Gini coefficient and poverty rate^28^.

Previous research on the relationship between the environment and disease outbreaks is undermined by two methodological flaws. First, the use of traditional linear regression models is somewhat to blame for the inconsistent results^29^. Such linear models do not take variable interactions, non-linearities, etc. into consideration, since they lack theoretical foundation. Data-driven machine learning techniques have been recently surfaced as approaches to address these shortcomings in environmental health research^30^. The random forest algorithm is an effective ensemble learning technique for both classification and regression issues^31^. Second, unlike ordinary least squares (OLS), the random forest technique is not dependent on restricted model assumptions.^32,33^, Third, global regressions models applied in a body of research require the estimated coefficients are spatially stationary- that is; they do not fluctuate throughout space regardless of location. This method is problematic, particularly when the study area is extensive^34^.

To address these failures and to investigate the interaction between neoplasms and some driving factors, our study aimed to (1) determine the spatial distribution and spatial clustering patterns of the age-adjusted neoplasms incidence rate in Iran, (2) demonstrate the provinces in which the interaction of high or low values of both dependent and explanatory variables are pronounced using popularity tool, (3) measure the spatial dependence of each explanatory variables as a function of the distance (4) assess the spatial relationships between the AAIR of neoplasms and various socioeconomic, environmental, and public health factors using MGWR and (5) evaluate the relative importance of independent variables on AAIR of neoplasms using Random Based Forest (RF).

## Methodology

### Topology of the study area

**Figure 1:**
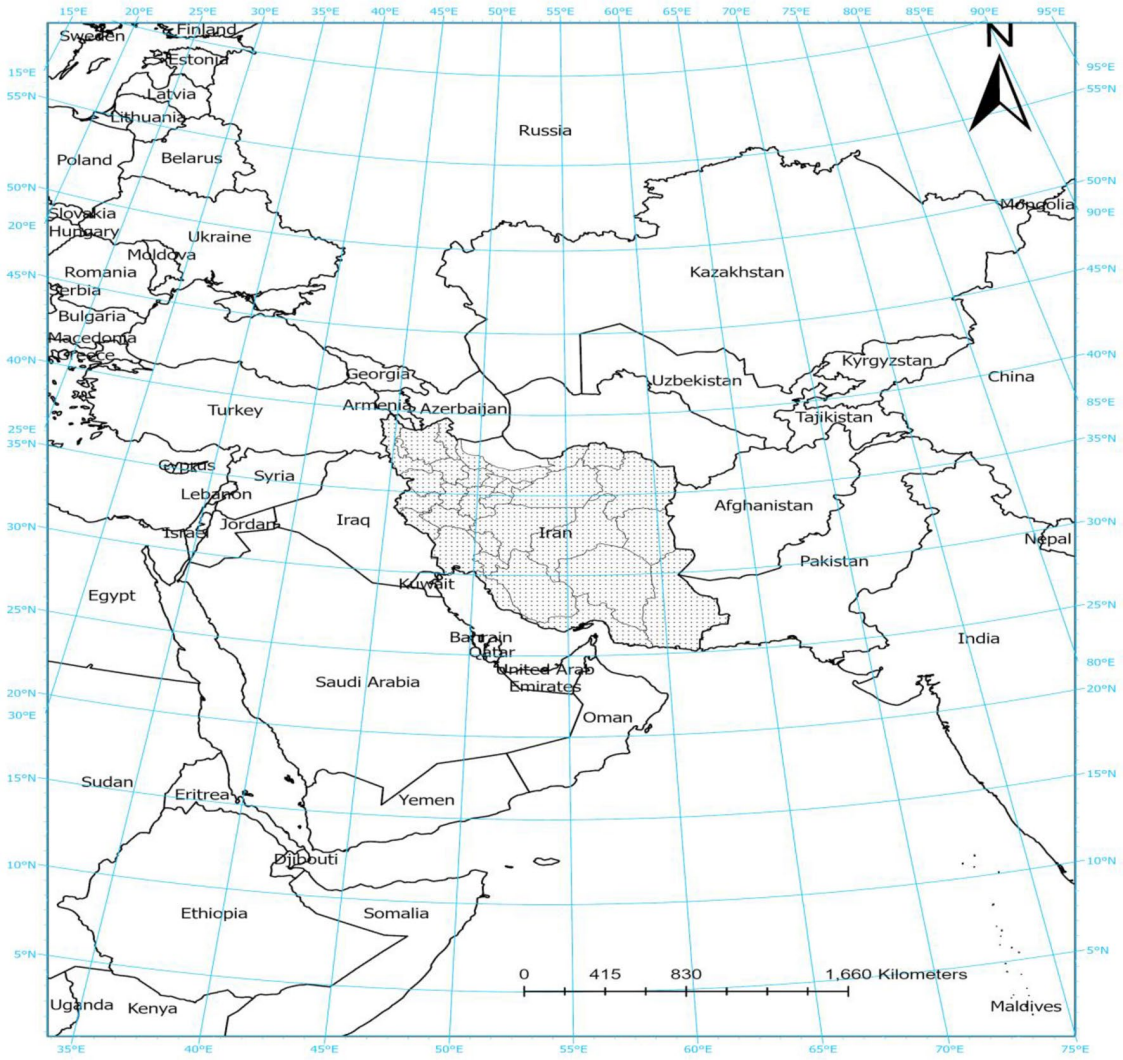
Study area and its location in the region. This map was generated using ArcGIS pro 2.5. (ESRI, Redlands, CA, USA, http://www.esri.com).

Iran is situated in the northern hemisphere above the equator, with its latitudinal location being 32.4279° N. Iran's coordinates are 53.6880° E, which places it in the eastern hemisphere. The country located in West Asia shares boundaries with the Gulf of Oman, the Persian Gulf, and the Caspian Sea (Fig. 1). Geographically speaking, it is mostly found on the Persian Plateau, encompassing 1,648,195 square kilometers (636,000 square miles) which makes it the sixteenth largest country in the world.

### Sources of data

Data on the age-adjusted incidence rate of neoplasms per 100,000 population for five years (2010, 2013, 2015, 2017, and 2020) and all 32 provinces were obtained from the Institute for Health Metrics and Evaluation (IHME, https://www.healthdata.org/research-analysis/gbd). Disability age-adjusted life years (DALYs) for Hepatitis B (HBV), Hepatitis C (HCV), and sexually transmitted diseases (STD) excluding HIV were obtained from the same source. Literacy rate, number of healthcare centers in each province, and Gini coefficients were collected from the Statistical Center of Iran (https://www.amar.org.ir/). Tobacco consumption rate (percentage of people who currently smoke one type of tobacco on a daily basis, %) were obtained from the National Institute of Health Researches of Iran (http://nihr.tums.ac.ir). Average annual UV index values^35^, and mean concentrations of ground-monitored PM_2.5_ (µg/m^3^)^36^ were collected from two recently published articles.

### Descriptive and exploratory analyses

#### Spatial autocorrelation and Hot Spot analyses

For an exploratory spatial study of our response variable (AAIR of neoplasms), we used the Moran's I statistic. Positive spatial autocorrelation is shown by a positive Moran's I value; negative spatial autocorrelation is indicated by a negative one, and values close to 0 reflect a spatially random pattern^37,38^. Four scenarios^37^ could occur in the local Moran's I index, which evaluates the relationship between points and neighbors; namely (High–High (H–H); indicates positive spatial autocorrelation for both the value of AAIR and its neighbors, High–Low (H–L), Low–High (L–H), and Low–Low (L-L)). Subsequently, an event's accumulation in very big or very small amounts can be examined using Getis-Ord Gi* index, which also includes markers of hot spots (high-risk areas) and cold spots (low-risk areas). Hot spots are indicated by positive Z-score values, and cold spots are indicated by negative Z-score values^39^. All the maps exhibited in this study were generated by ArcGIS pro 2.5.

#### Semivariogram

Semivariograms were used to determine a variable's spatial coherence. When two specimens are spatially coherent, it indicates that they are dependent on one another up to a predetermined distance. This dependency is thought to be represented by a semivariogram, a mathematical model^40^. Semovariogaram was calculated based on Eq. (1).

1 $$\gamma \left(h\right)=\frac{1}{2N(h)}\sum_{i=1}^{N(h)}{[Z({x}_{i}-Z\left({x}_{i}+h\right)]}^{2}$$

where, *N* (*h*) is the value of the semivariogram for distance *h*, *Z*(*x*_*i*_) denotes the sample value at point *x*_*i*_, and *Z*(*x*_*i*_ + *h*) is the sample value at point *x*_*i*_ + *h*. The symbol (*h*) indicates the distance in the designated direction between the position *x*_*i*_ and *x*_*i*_ + *h*.

Range, sill, and nugget are three parameters of a semivariogram, which are defined as follows:

Range or impact radius is the length of time that the variogram takes to approach the horizontal line and reach a fixed point.

Sill is the variogram's consistent value within the effect's range. Its value is the sum of the variances of all the samples that was utilized to determine the change in facade.

Nugget; for *h* = 0, it is the variogram's value at the origin. Its value ought to be zero in theory^41^.

Using the spatial dependence index based on Eq. (2), the optimal correlation is found.

2 $$SD=\frac{nugget}{nugget+partialsill} \times 100$$

Three scenarios are considered while examining the value of SD: less than 25% indicates strong geographical correlation; between 25 and 75% demonstrate moderate spatial correlation; and more than 75% depicts poor spatial correlation^42^.

#### Multi-scale geographically weighted regression

MGWR can be used to investigate correlations between explanatory and dependent variables that differ spatially. It includes both the recently introduced Multiscale GWR (MGWR) technique, which relaxes the assumption that all of the processes being modeled run at the same spatial scale, and the previously used Geographically Weighted Regression (GWR) approach to modeling process spatial heterogeneity^43^. In order to evaluate geographical variation in the relationship between socioeconomic traits, environmental factors, and neoplasm incidence rates, we used MGWR modeling. As opposed to classical regression, this method permits the model parameters to change in different geographic units. We chose the fixed Gaussian spatial kernel in the MGWR model to control for an ideal bandwidth, which is thought to be constant throughout space^43^.

The bandwidth search was conducted using the Golden Section search option, which reduces the range of values for the ideal bandwidth and delivers the lowest score^44^. For the optimization criterion, we employed the corrected Akaike's Information criterion (AICc), whereby the bandwidth with the lowest AICc is chosen and applied to the analysis. MGWR analysis was conducted in MGWR 2.2 software^45^.

#### Popularity analysis

Popularity tool counts the instances of the input raster values at each position and assigns an ordinal ranking to each one, meaning the most, second, and so on are the most popular.^46^. In other words, this tool finds, cell by cell, the value in an argument list that is at a particular degree of popularity. To prepare the maps of bivariate interaction of each two variables (dependent and one independent) and analyze high-high or low-low interaction effects in each province, raster maps of AAIR of neoplasms and each covariate values were first prepared. Thereafter, each raster was categorized to 10 classes and each class was numbered from 1 to 10 to classify values from low to high. Then, the interactions between each of variables were tested by popularity analysis tool using ArcGIS pro2.5. It is worth nothing that two covariates, namely the number of healthcare centers and literacy rate in each province which assumes to be inversely correlated with AAIR, were conversely ranked.

#### Random forest and validation

A regression-based method according to ensemble learning is called a random forest (RF)^47^. Many regression trees that have grown to their maximum size without pruning make up the algorithm. The average of the forecasts from each individual tree yields the final predictions. The RF, in contrast to standard regression, does not rely on rigorous statistical assumptions, models complicated correlations, and takes variable interaction into account. A series of trees based on *N* independent observational data were used to create an RF. Following the construction of each tree, the test data are added and the number of trees corresponding to the input vector and output is defined. Calculating the final outcome involves averaging these outputs. The default value for the number of trees parameters is 100. It has been asserted that increasing the number of trees in the forest model will result in more accurate model prediction^48^. Accordingly, we increased the number of trees to 500, and 5 randomly sampled variables at each split were suggested to have high prediction accuracy^49^. To analyze the performance metrics of RF model for both training and validation data, we used coefficient of determination (*R*^2^) and standard error (*SE*). In order to link prediction with the assignment of relevance to specific predictors, importance measures have been developed to characterize the contribution of explanatory variables to prediction^50,51^; which was described by Efron^52^. Here, we examined the arrangement of variable importance (VIMP), which is widely used as a method to improve the interpretability of Random Forests.

Importance was calculated using Gini coefficient, which can be thought of as the number of times a variable is responsible for a split and the impact of that split divided by the number of trees. (https://pro.arcgis.com/en/pro-app/3.1/tool-reference/spatial-statistics/how-forest-works.htm).

## Results and discussion

### Spatial distribution of the age-adjusted incidence rate of neoplasms

**Figure 2:**
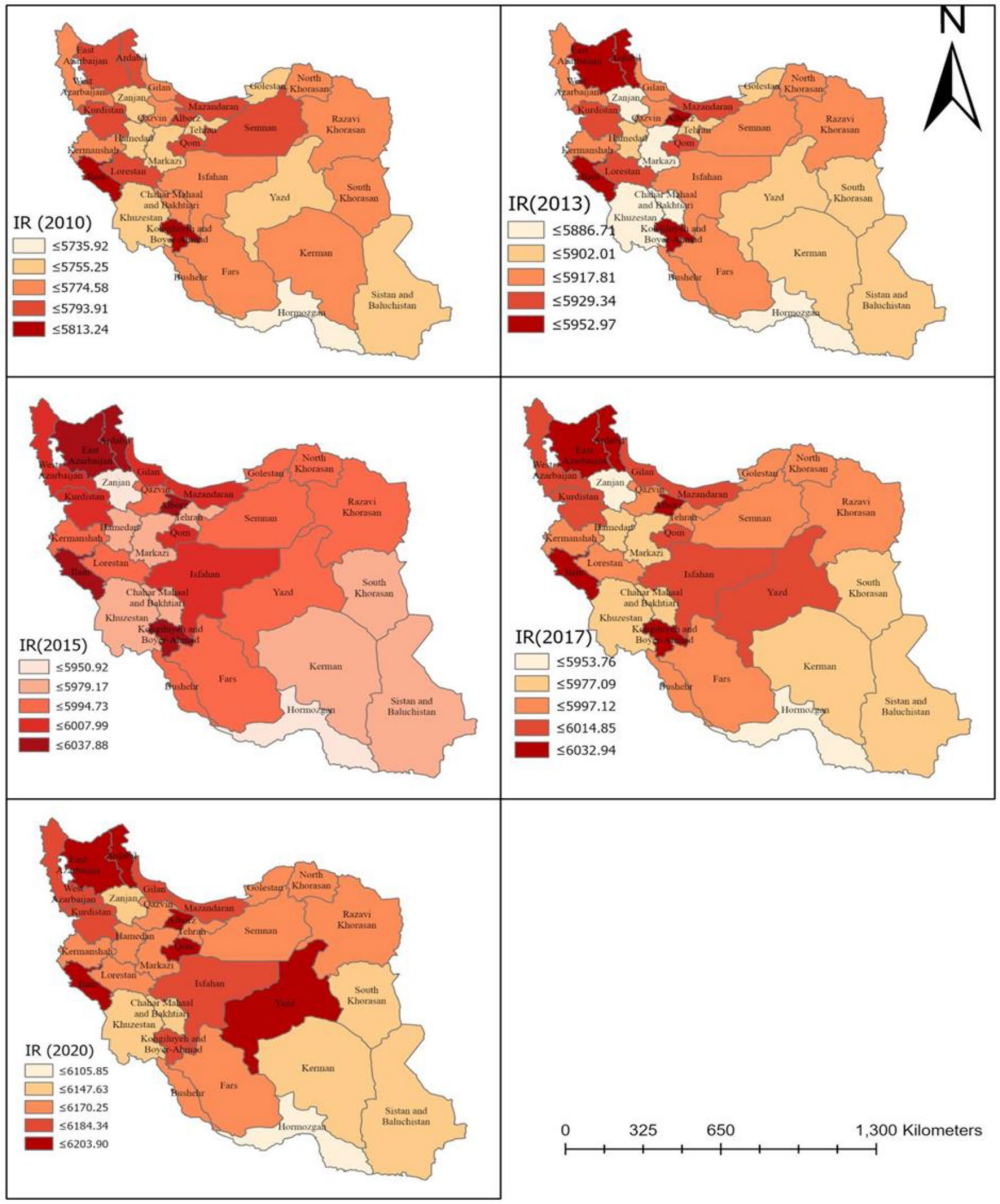
Geographic distribution of the Age-Adjusted Incidence Rate (AAIR) of Neoplasms per 100,000 people at the province level in 2010, 2013, 2015, 2017, and 2020. This map was generated using ArcGIS pro 2.5. (ESRI, Redlands, CA, USA, http://www.esri.com).

The age- adjusted incidence rate of neoplasms for each county in terms of each year throughout the study period is displayed in Fig. 2. As can be seen, the distribution was found to be heterogeneous. The average rates were 5765.16, 5909.7, 5989.85, 5992.39 and 6265.92 (new cases/100,000 population) for 2010, 2013, 2015, 2017, and 2020, respectively. Greatest rate in 2010 was observed for Kohgiluyeh and Boyer-Ahmad, and Ilam provinces, which are located in the west part of the country; whereas, the lowest AAIR belonged to Hormozgan in the same year. A significant increase in rate was noticed in 2013, mainly in the northwest and west of the country, where Kohgiluyeh and Boyer-Ahmad, Ilam, Qom, Ardabil, and East Azerbaijan recorded the highest values. A shift was also notified in 2015 and the same provinces recorded the highest rates. With the exception of Zanjan, a cluster of elevated rate was observed in 2015, 2017 and 2020, extending from northwest into central parts of Iran. The number of cases was remained almost the same in 2017 as compared with 2015, and the pattern was somewhat similar to that of 2015. Incidence of neoplasms was highest in 2020 over the course of the 5-year period. It is worth mentioning that cases in Yazd province—in the central part of country—which had remained low during 2010 and 2013, gradually increased in 2015 and 2017 and reached the highest value in 2020. Furthermore, Ilam took priority over other provinces in terms of incidence rate for three consecutive periods of study (2010, 2015, and 2017); though, East Azerbaijan showed the highest incidence rate in 2020. Overall, annual incidence rate have been increasing since 2010, and the number of reported cases of neoplasms has remained high. It is anticipated that in the Islamic Republic of Iran, there will be 160,000 new cases of cancer by 2025, in excess of 112,000 instances that were reported in 2016. This is a 42.6% rise, of which variations in risk and population structure were ascribed to 13.9% and 28.7% of the increase, respectively^53^. Regarding certain cancer subtypes, thyroid cancer (113.8%), prostate cancer (66.7%), female breast cancer (63.0%), and colorectal cancer (54.1%) are expected to see the most increases in the number of new cases^53^. In 2016, stomach, colorectal, and breast cancers were the most prevalent cancer kinds in the nation; by 2025, it is expected that these three cancer types will still be in the lead^53,54^. In a comprehensive study conducted by Khanali and Kolahi^55^, it was reported that between 2000 and 2016, the incidence of cancer has almost been doubled; its age-standardized rate increased less sharply, though. In certain regions of the nation with high incidence rate, specific tumors showed a distinct distribution pattern^55^. The Islamic Republic of Iran's rising trends in the incidence of the most prevalent cancer types highlight the necessity of developing and implementing national cancer control initiatives that are specifically suited to each region of the nation.

### Spatial distribution of explanatory variables

Spatial distribution of independent variables is shown in Supplementary Fig. S1 (Supplementary Information). At the national level, DALYs of STD varied more than three times, from 11.32 in South Khorasan to 32.21 per 100,000 in Kohgiluyeh and Boyer-Ahmad, indicating major differences between provinces. DALYs of HBV and HCV are distributed unevenly across provinces in Iran. There are regional similarities in the HBV and HCV distribution features (Supplementary Fig. S1). The highest values for both HBV and HCV were observed for Golestan, Qazvin, Hamedan, Fars, and Kermanshah. Tobacco consumption rate were prevalent in Qazvin, Fars, Bushehr, and West Azarbaijan. There was a noticeable concentration in the distribution of UV index among the provinces. While the southern parts of the country had the highest values of UV index, the west regions including Ilam. Khuzistan, and Kohgiluyeh and Boyer-Ahmad had the most substantial annual mean concentrations of PM_2.5_. A yearly annual mean concentration of 5 µg/m^3^ for PM_2.5_ values is the recommended threshold by WHO^56^. However, none of the studied areas adhered to this limit and all of them exceeded the suggested threshold, indicating 22–52 times higher than that of recommended by WHO. Except for Bushehr, people with limited literacy rate were concentrated in regions in the west and southwest.

The Gini coefficient calculates the deviation from perfect equality in the distribution of income (or, occasionally, consumption expenditure) among people or households in an economy. Gini coefficient was the highest in Sistan and Baluchestan showing income inequality across the population living in this area. Ardabil and Qazvin recorded the lowest values indicating more equal distribution of wealth in these provinces. There was a remarkable difference in the number of healthcare centers distribution across regions of Iran where the lowest numbers were observed in Alborz, Qom, Markazi, and Fars.

### Assessment of global Moran's I

**Figure 3:**
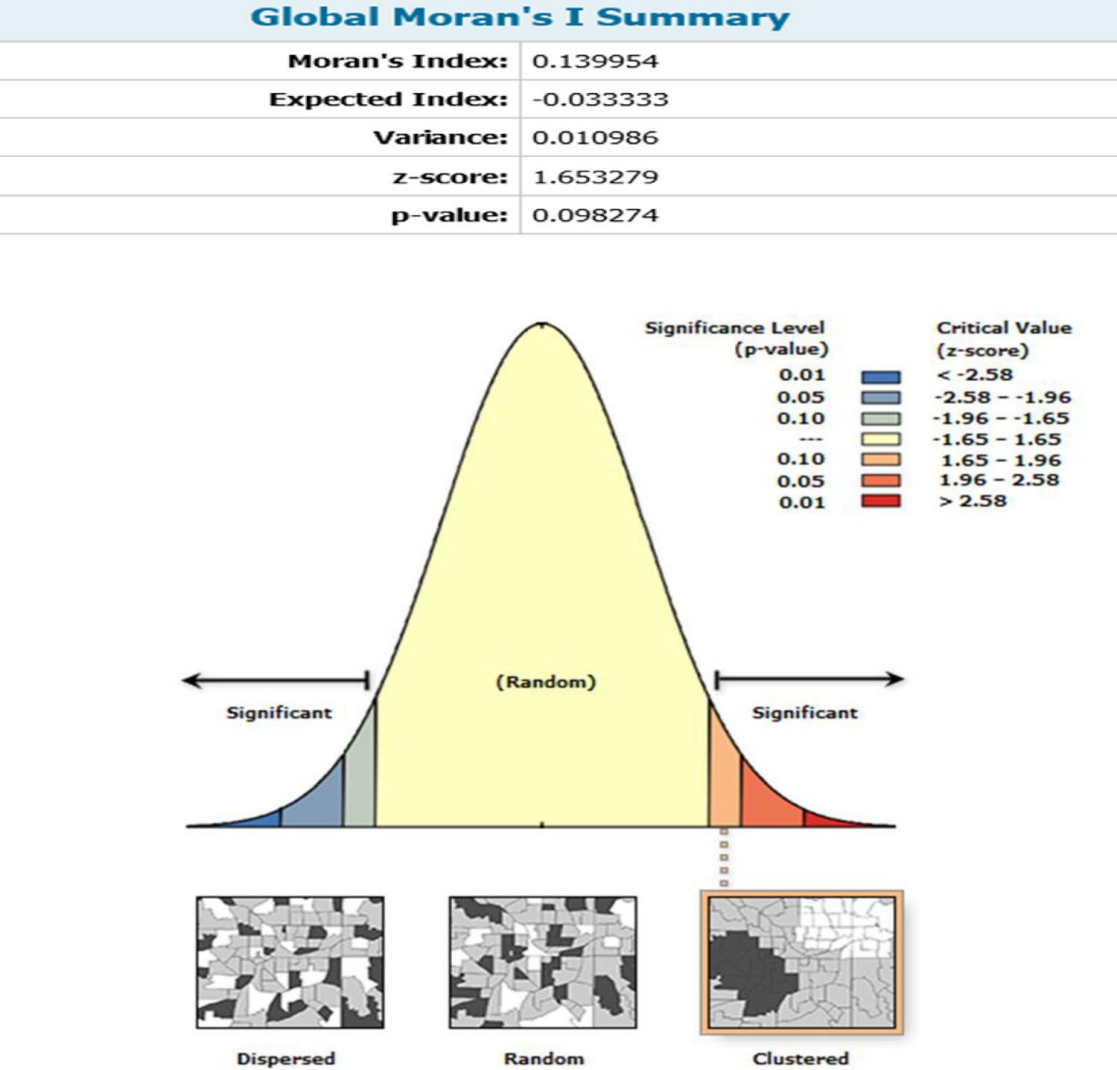
Spatial autocorrelation of the age-adjusted incidence rate of neoplasms in 2020.

Spatial dependence can be established with particular statistical measures. In this model, the question of whether neoplasms incidence rate in close proximity to one another were more similar than those in remote proximity was investigated using spatial autocorrelation. The feature locations and assigned values were used to assess the AAIR spatial autocorrelation results prior to the application of hot spot analysis (Fig. 3). As depicted, the incidence rate of neoplasms in the study area was in the clustering pattern in 2020, according to the geographical autocorrelation (global Moran's I) over the study period. The corresponding *p*-value was less than 0.1, with a Moran's index of 0.139. Based on these findings, the equivalent Z-score was 1.652, which implies small significant clustering patterns of data and the probability that this clustering pattern due to the product of chance was less than 10%. For other study period years (2010, 2013, 2015, and 2017), however, Z-score ranged from − 1.65 to + 1.65 (includes zero), which indicates that the random pattern could describe AAIR data for those years and there was no clustering pattern (Supplementary Fig. S2). Olfatifar et al., investigated the patterns of clustering in the incidence rate of breast cancer in Iran between 2004 and 2010. They found that the Moran's index for 2004 was positive and significant, indicating that the cancer incidence pattern was clustered and that the provinces or blocks had values comparable to those of their neighbors. However, the index was not significant for the other years of study^57^.

### Hotspot inspection

**Figure 4:**
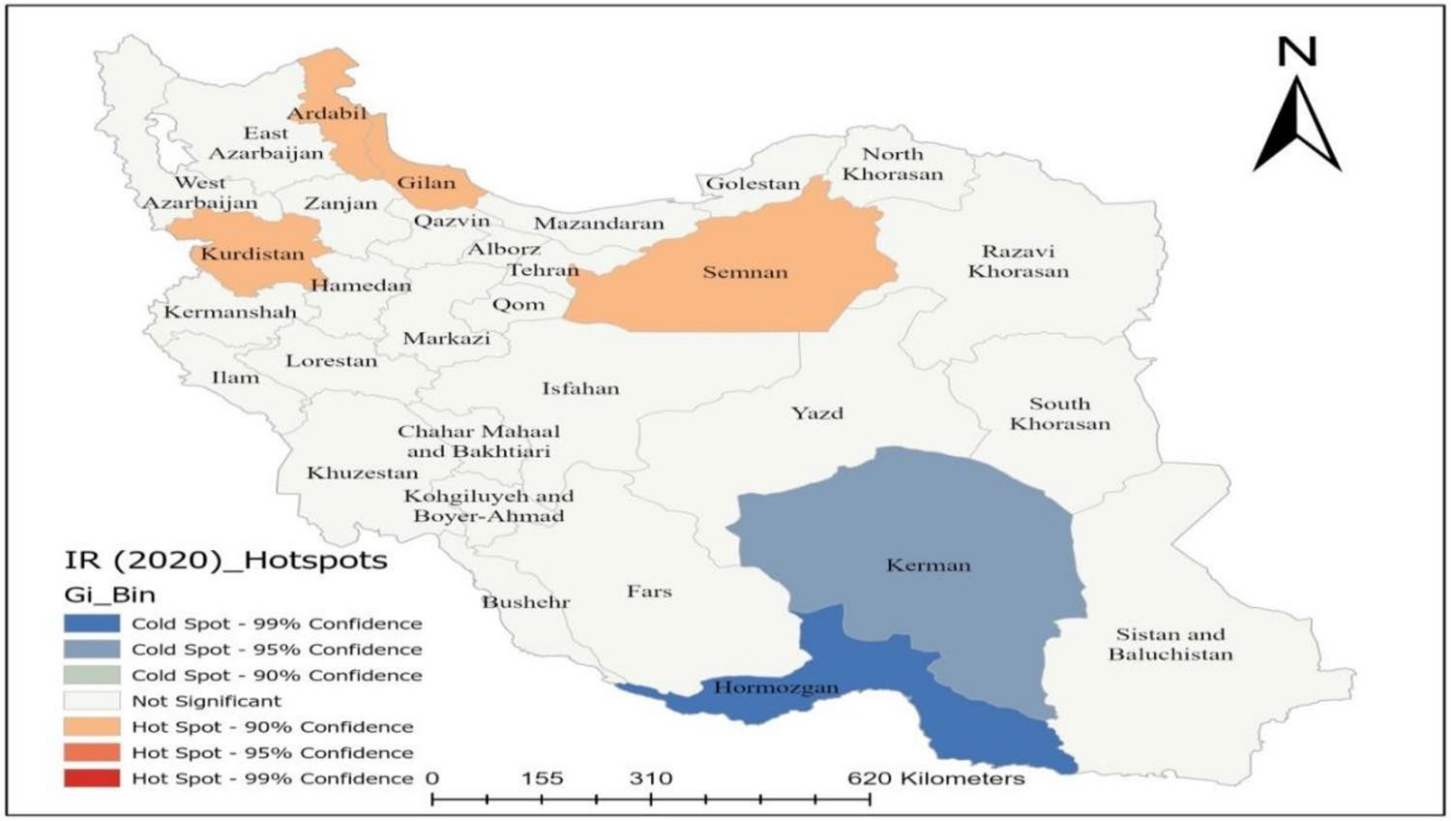
Hotspot map of AAIR of neoplasms in 2020. This map was generated using ArcGIS pro 2.5. (ESRI, Redlands, CA, USA, http://www.esri.com).

The benefit of hotspot analysis from the standpoint of disease prevention and control and policy-making is its ability to characterize particular geographic units based on statistically significant differences^58^. The Getis-Ord Gi* statistic was used to find neoplasms hotspots, and the outcomes are shown in Fig. 4. With a 90% confidence level, this figure shows the clustering of high values (hot spots) for Semnan, Kurdistan, Gilan, and Ardabil. As depicted, this high-high clustering suggests that in these areas environs are more likely to see high values of AAIR (H–H). Additionally, with 95% confidence level low values (cold areas) were detected two provinces located in the southern part of the country. Hot spot analysis, also known as Getis-Ord GI*, has been used to predict multiple disease outbreaks in the past and is seen to be a useful method for identifying spatial clusters of both high and low values^38,59,60^. After determining which counties are within the hot areas, precautions should be taken to stop the rising trend to nearby nations, particularly those that are in cold spots^61^.

### Multiscale geographically weighted regression

The summary statistics of the calculated coefficients of the local terms (MGWR model) are shown in Table 1. The mean annual UV index has a statistically significant result (p < 0.05) according to the geographical heterogeneity test, indicating spatial diversity in this variable. With the lowest AIC value (~ 95) and the highest R^2^ value (0.38) and adjusted R^2^ of 0.23, the MGWR model accounted for almost 38% of the variation in the incidence rate of neoplasm among Iranian population. It is clear from Table 1 that that except for UV index other variables were not associated with AAIR of neoplasms (*p* > 0.05).

**Table 1:** Summary of MGWR estimation relating nine explanatory variables.

Explanatory variable	Mean (STD)	Median	p values
Intercept	0.000 (0.002)	− 0.001	0.800
PM 2.5 (µg/m^3^)	− 0.057 (0.001)	− 0.057	0.780
DALYS (STD)	0.168 (0.002)	0.167	0.405
DALYS (HBV)	0.634 (0.002)	0.634	0.520
DALYS (HCV)	− 0.890 (0.002)	− 0.890	0.421
Number of healthcare centers	− 0.024 (0.002)	− 0.023	0.901
Tobacco consumption rates (%)	0.052 (0.002)	0.052	0.782
UV index	− 0.531 (0.003)	− 0.530	0.025
Literacy rate (%)	0.112 (0.000)	0.112	0.630
Gini coefficient	− 0.228 (0.001)	− 0.228	0.328
Model diagnostics			
AIC:93.265	AICc: 109.159	R^2^:0.378	Adjusted R^2^:0.230

The negative coefficient observed for this variable indicates it’s inversely association with AAIR of neoplasms. In a very recent study conducted by Gregoire et al., exposure to UV rays was broken down into quintiles and the authors concluded that there was no correlation between UV exposure and the overall risk of breast cancer. Higher UV exposure, however, was inversely linked to a lower incidence of one breast cancer type (without estrogen receptors (ER)^62^.

### Semivariogram analysis of covariates

**Figure 5:**
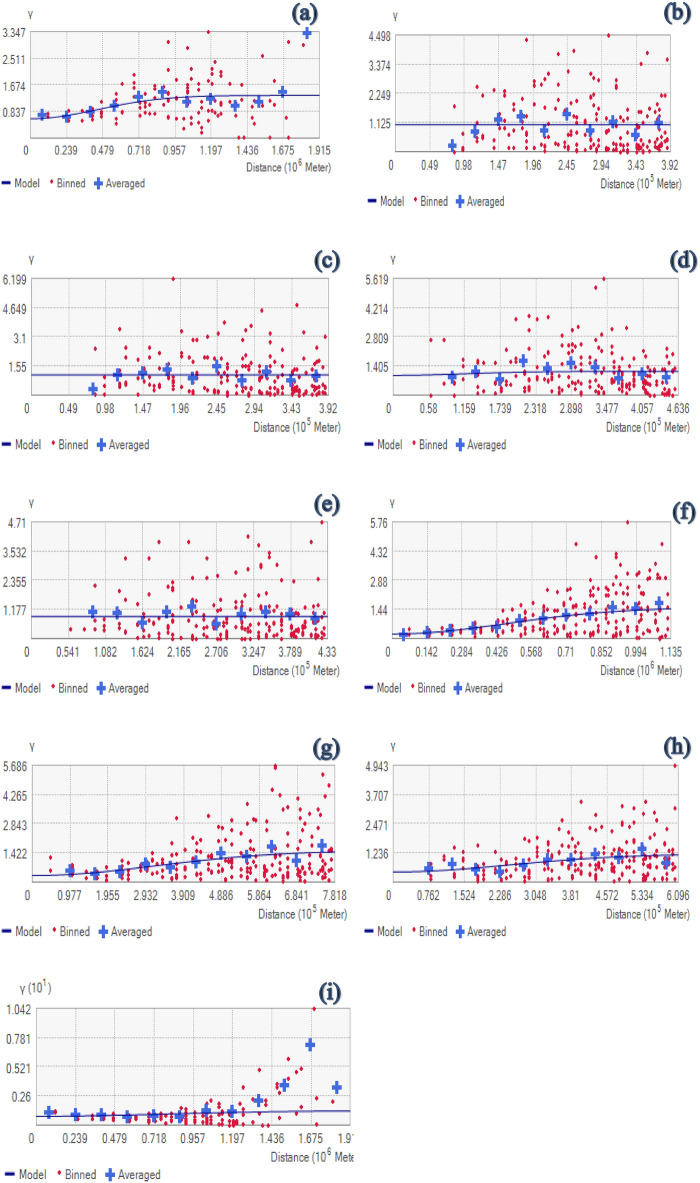
Result of semivariogram. (**a**) DALYs of STD, (**b**) DALYs of HBV, (**c**) DALYs of HCV, (**d**) Number of healthcare centers, (**e**) Tobacco consumption rate (%), (**f**) UV index, (**g**) Annual mean concentration of PM_2.5_, (**h**) Literacy rate (%), and (**i**) Gini coefficient.

Semivariogram findings are displayed in Table 2 and Fig. 5. The highest value of nugget, as a key parameter of the semivariogram, was associated with DALYs of HBV, DALYs of HCV, and the number of healthcare centers, while its lowest value is correlated with literacy rate, and PM_2.5_. Again, as can be seen, the lowest value of Semivariogram range was meaningfully associated with HBV, HCV, the number of healthcare centers and tobacco consumption rate, while its highest value was correlated with UV index, and PM_2.5_. The sill's results indeed reveal that its lowest value is associated with HBV, HCV, tobacco consumption rate, while its maximum value is related to PM _2.5_ and UV index. According to the SD (%) results, PM_2.5_ and UV index exhibited strong spatial correlation (SD < 25%); while STD, literacy rate and Gini coefficients showed moderate spatial correlation over the entire area of Iran (25% < SD < 75%). Other criteria including HBV, HCV, number of healthcare centers, and tobacco consumption rate demonstrated weak spatial correlation (SD > 0.75%).

**Table 2:** Results of the semivariogram's parameters.

Criterion	Nugget	Range	Partial Sill	SD
DALYs of STD	0.610	1,087,381.24	0.730	45%
DALYs of HBV	1.045	391,967.98	0	100%
DALYS of HCV	1.083	391,967.98	0	100%
Number of healthcare centers	0.952	284,720.86	0.196	83%
Tobacco consumption rate	0.902	432,984.63	0	100%
UV index	0.223	94,624.41	1.302	14%
PM2.5	0.311	781,812.14	1.202	20%
Literacy rate	0.425	609,603.85	0.753	35%
Gini coefficient	0.790	1,914,693.89	0.495	61%

### Detecting regions with elevated incidence rate of neoplasms that are located in critical values of explanatory variables

It is of paramount importance to exhibit in which provinces higher or lower values of each independent variable affect the neoplasms rate. To address this question, on a cell-by-cell basis, the popularity tool in ArcGIS pro 2.5 was used to identify the value in an argument list (covariates) that is at a particular level of popularity (incidence rate of neoplasms). The findings of the examination of each covariate on values of AAIR of neoplasms in each cell are shown in Supplementary Fig. S3(1)), which depicts the areas where higher incidence rate of neoplasms were observed (based on the DALYs of STD). It is clear that the higher DALYs of sexually transmitted diseases (excluding HIV) in Kohgiluyeh and Boyer-Ahmad and Sistan and Baluchistan have contributed to the shifted values of AAIR of neoplasms.

The counties in the south and northwest were mostly affected by DALYs of HBV and HCV determinants; the center (Isfahan) was also affected by both covariates (Supplementary Fig. S3(2), (3)). Similar to HBV, HCV was more significant in Bushehr, Alborz, Golestan, Gilan, Ardabil, and west Azerbaijan. Seemingly, the reduced number of healthcare centers has taken part in increased AAIR of neoplasms in Qom and Qazvin (Supplementary Fig. S3(4)), while higher rate of tobacco consumption was found as a significant factor in determining the incidence of neoplasms in Qazvin and Bushehr (Supplementary Fig. S3(5)). AAIR of neoplasms is considerable in the South and moderate in the central parts of the country, but less significant in the north when it comes to underlying UV index (Supplementary Fig. S3(6)). In the meantime, counties in the Southwest have the highest rate of AAIR due to the detrimental effects of PM_2.5_ (Supplementary Fig. S3(7)). There has been a high concentration of PM_2.5_ in the provinces of Khuzestan, Ilam, Kermanshah, Lorestan, Chaharmahal and Bakhtiari, Fars, Bushehr, Tehran, and Isfahan in recent years^36^, which could be assigned to natural dust storms. Indeed, ineffective ambient air pollution abatement policies at the regional, national and subnational levels in Iran, plus ongoing urbanization and industrialization and associated emissions, as well as unsustainable development have led to annual level of PM_2.5_ concentration more than WHO AQGs standards^63,64^. Dust storm events, especially during the spring, have deteriorated the air quality in western and south-western provinces as compared to other regions. Sistan and Baluchistan severely showed higher rate of AAIR due to the low literacy rate of its community followed by Kerman, Hormozgan, Khuzestan, Ilam and East Azerbaijan (Supplementary Fig. S3(8)) similarly, AAIR of neoplasms was also more significant in Sistan and Baluchestan as a result of higher Gini coefficients followed by Tehran and Hamedan (Supplementary Fig. S3(9)). The Gini coefficient calculates how far an economy's income or consumption expenditures are distributed from a perfectly equal distribution among its dwellers or households (https://databank.worldbank.org/metadataglossary/world-developmentindicators/series/SI.POV.GINI).

While it has infrequently been applied in epidemiology and medicine, Gini coefficient which considers the absolute relative difference between people, accurately captures the variability in illness risk ^65,66^.

### Random forest

**Figure 6:**
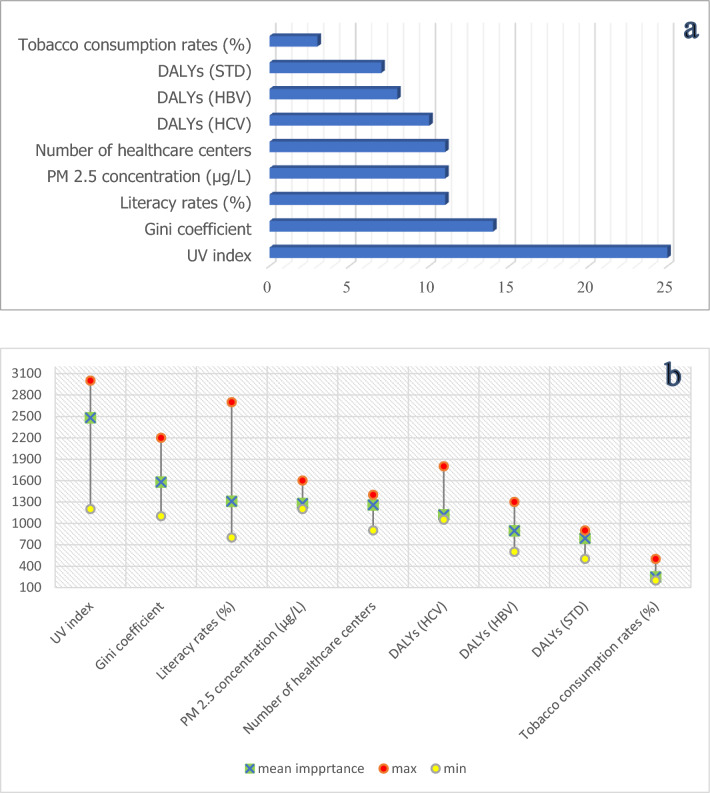
(**a**) VIMP (%) obtained based on RF. The proportion of the overall sum of the Gini coefficients is indicated in the % (x axis of (**a**)), (**b**) Max, Min and Mean of each studied VIMP Based on RF through 500 trees. The Gini coefficients for each listed variable are added together in the Importance axis (Y in (**b**)).

The benefits of the Random Forest technique are flexibility in data types, minimal sensitivity to spatial autocorrelation, and multicollinearity^67,68^. The significance of determinants described by the percentage, utilized in the RF model was ranked in Fig. 6a. Figure 6b illustrate the ranges (distribution) of VIMP (including min, max and mean) through these 500 trees. The findings show that UV index, followed by Gini coefficient, literacy rate, annual mean concentration of PM_2.5_, and the number of healthcare centers were the most driving factors in explaining the prevalence of neoplasms; whereas, tobacco consumption rate played a negligible role. In RF model, a continuous variable is predicted by comparing the observed value of each test feature with the predictions made by the trained model; Hence, we examined the validation of model by *R*^2^ and SE for both training and validation of data (Table 3). As depicted, the high correlation coefficient (*R*^2^ > 0.9) and low SE for both training and validation procedure imply the accuracy of the model. Additionally, in statistics, the low *p* value (< 0.05) indicates the suitability of the model. The training and validation share defined as the percentage of overlap between the ranges of training data and the input explanatory variable, as well as the percentage of overlap between ranges of the validation data and the training data were also examined for validation of model (Table 4). The training share column shows the proportion of overlap between the values of all the features in the input training features and the values of the training subset for each continuous explanatory variable. It is clear that for all explanatory variables above 95% of the range of values of the input training features are covered by the training dataset which is quite satisfactory. To generate the validation share diagnosis, a comparable calculation was thence carried out (Table 4). The high values of the shared UV index, DALYs (HBV), DALYs (HCV), and Tobacco consumption rate (%) indicate the overlap occurred between the validation subset for each of these variables. The training subset shows the importance of them in predicting the AAIR of neoplasms in each province.

**Table 3:** Results of the parameters used for the validation of model.

	R^2^	SE	p_value_
Training data	0.917	0.036	< 0.001
Validation data	0.990	0.007	0.028

**Table 4:** Training and validation share of each determinants used in FR.

Criterion	Training share	Validation share
UV index	0.961	0.891
Gini coefficient	0.950	0.332
Literacy rates (%)	1	0.305
PM 2.5 concentration (µg/L)	0.983	0.283
Number of healthcare centers	1	0.114
DALYs (HCV)	1	0.600
DALYs (HBV)	1	0.692
DALYs (STD)	1	0.143
Tobacco consumption rates (%)	1	0.411

## Conclusions

In Iran, as a middle income country in middle-east, the incidence rate of neoplasms has substantially increased from 2010 to 2020. Small significant clustering patterns of AAIR of neoplasms were noticed in 2020. By using MGWR, we discovered that AAIR of neoplasms was significantly and conversely correlated with UV index values. The SD (%) results obtained from semivariogram analysis, however, revealed that despite DALYs of STD, literacy rate, and Gini coefficient had moderate spatial correlation throughout the country, PM_2.5_ and UV index showed strong spatial association. Weak spatial correlation was shown by other criteria, such as DALYs of HBV, HCV, the number of healthcare centers, and tobacco consumption rate. The provinces that are heavily affected by each explanatory variable were identified using popularity tool. Additionally, the results highlighted the good accuracy of RF model in simulating neoplastic regions. Furthermore, UV index and Gini coefficient were the two most influential factors according to our nationwide assessment of the variable importance.

### Limitations

Given its limitations, the results of our investigation should be taken into consideration. Notably, our study did not take into account critical environmental elements that may have an impact on incidence rate of neoplasms, such as Air Quality Index (AQI), Organic and inorganic chemicals (i.e., gasoline, asbestos). Likewise, we were unable to include family history, diet and obesity, and psychological factors which are linked to incidence of cancers, in our analysis, due to the lack of credential data for some provinces. Additionally, we considered the DALYS of HVB and HVC for the risk factors of all neoplasms in Iran owing to the few articles that have established the relation of infection with one or both hepatitis virus as a risk factor of other type of cancers (including bile duct cancer and lymphoma) in addition to liver cancer. Similarly, it is approved that STD can enhance the risk for developing certain cancers besides cervical cancer. Despite the insignificant and subtle correlation here observed between AAIR of neoplasms and these variables, our finding raise concerns about the importance of these factors on each cancer type and highlight the importance of evaluating the spatial effects of being afflicted with some of these specific infections and developing certain cancers.

### Supplementary Information

Supplementary Figures.

## Data Availability

Data will be made available from the corresponding author on reasonable request.
