# Supplementary material for: Impacts of socioeconomic and environmental factors on neoplasms incidence rates using machine learning and GIS: a cross-sectional study in Iran
Source: Sci Rep. 2024 May 8;14:10604. doi: 10.1038/s41598-024-61397-5 (PMC11078954; doi:10.1038/s41598-024-61397-5)
Supplement: Supplementary file 1 — Supplementary Figures. [file 41598_2024_61397_MOESM1_ESM.docx]

**Supplementary**


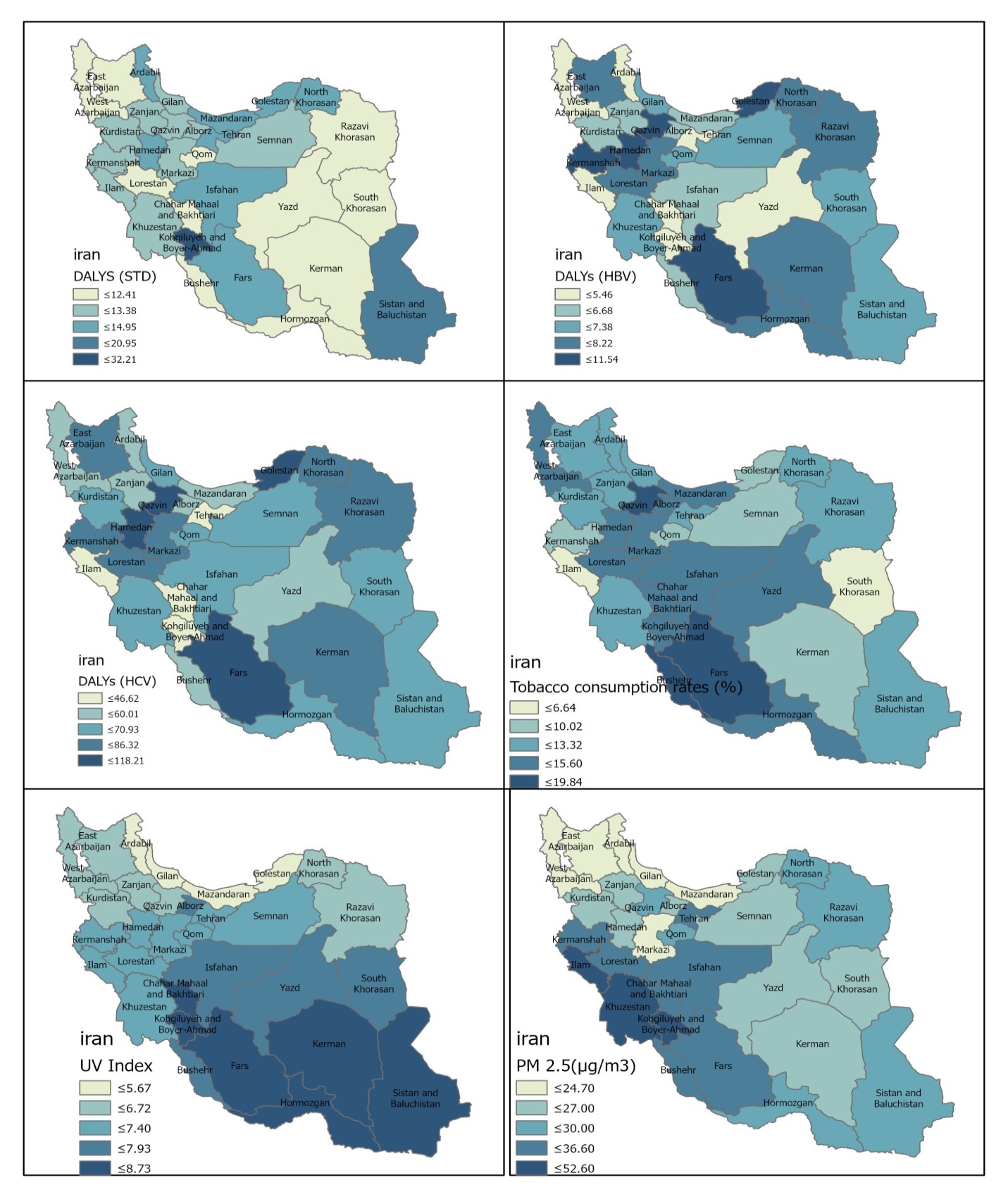


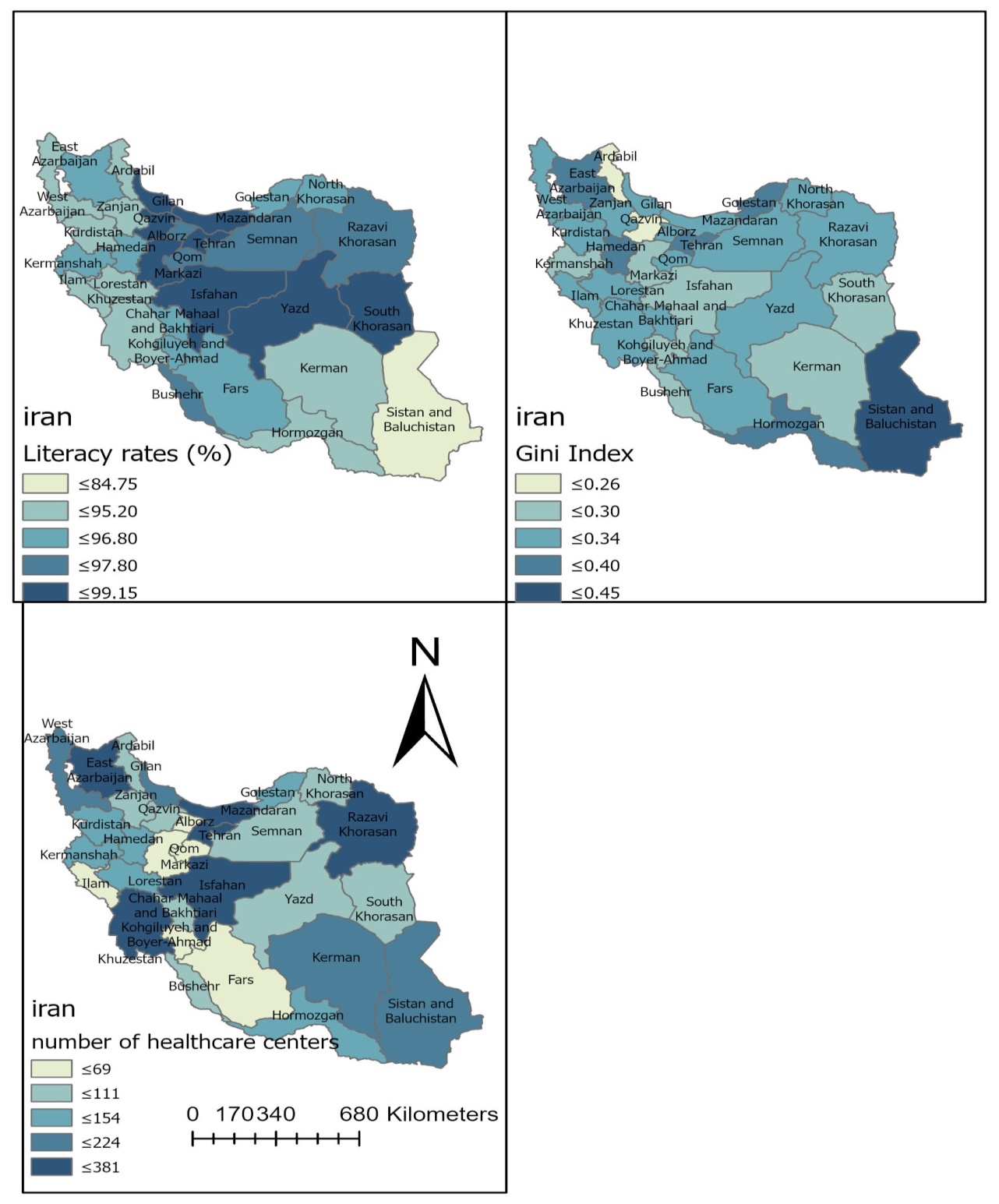


Fig. S1. Spatial distribution of the nine independent variables at the province level; (a) DALYS(STD), (b) DALYS(HBV), (c) DALYS (HCV), (d) Tobacco consumption rates (%), (e) UV index, (f) Annual mean concentration of PM _2.5_, (g) Literacy rate (%), (h) Gini coefficients, (i) Number of healthcare centers. This map was generated using ArcGIS pro 2.5. (ESRI, Redlands, CA, USA, http://www.esri.com).

| 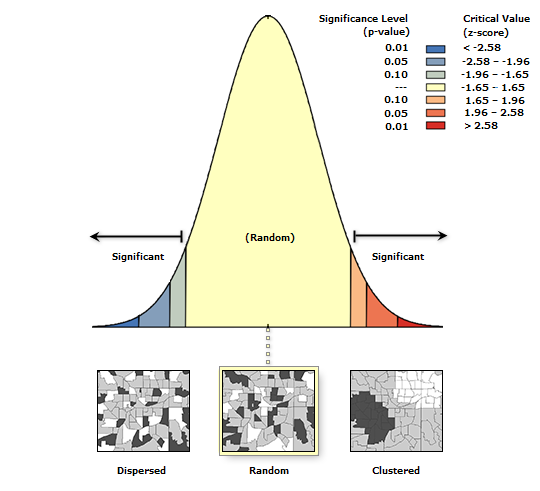  b  a | 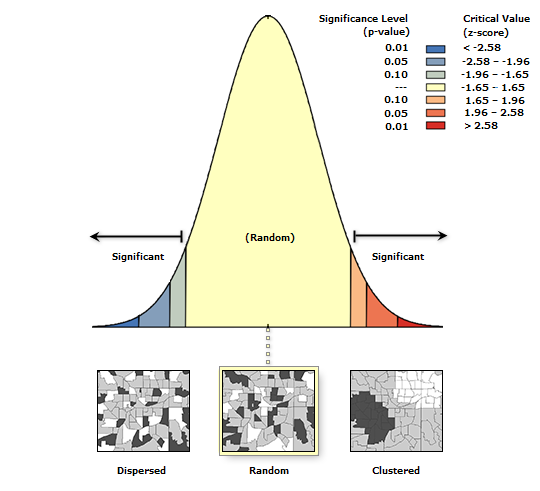 |
| --- | --- |
| 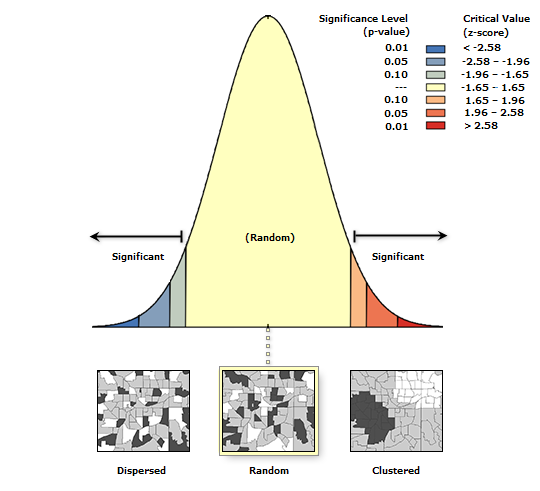  d  c | 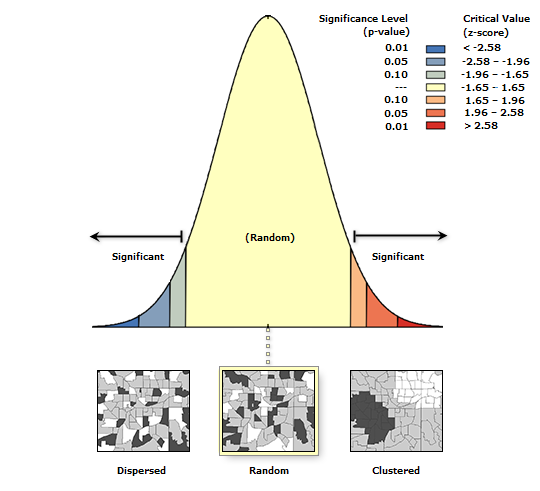 |

Fig.S2. Spatial autocorrelation of AAIR of neoplasms in Iran; in (a) 2010, (b) 2013, (c) 2015, (d) 2017.


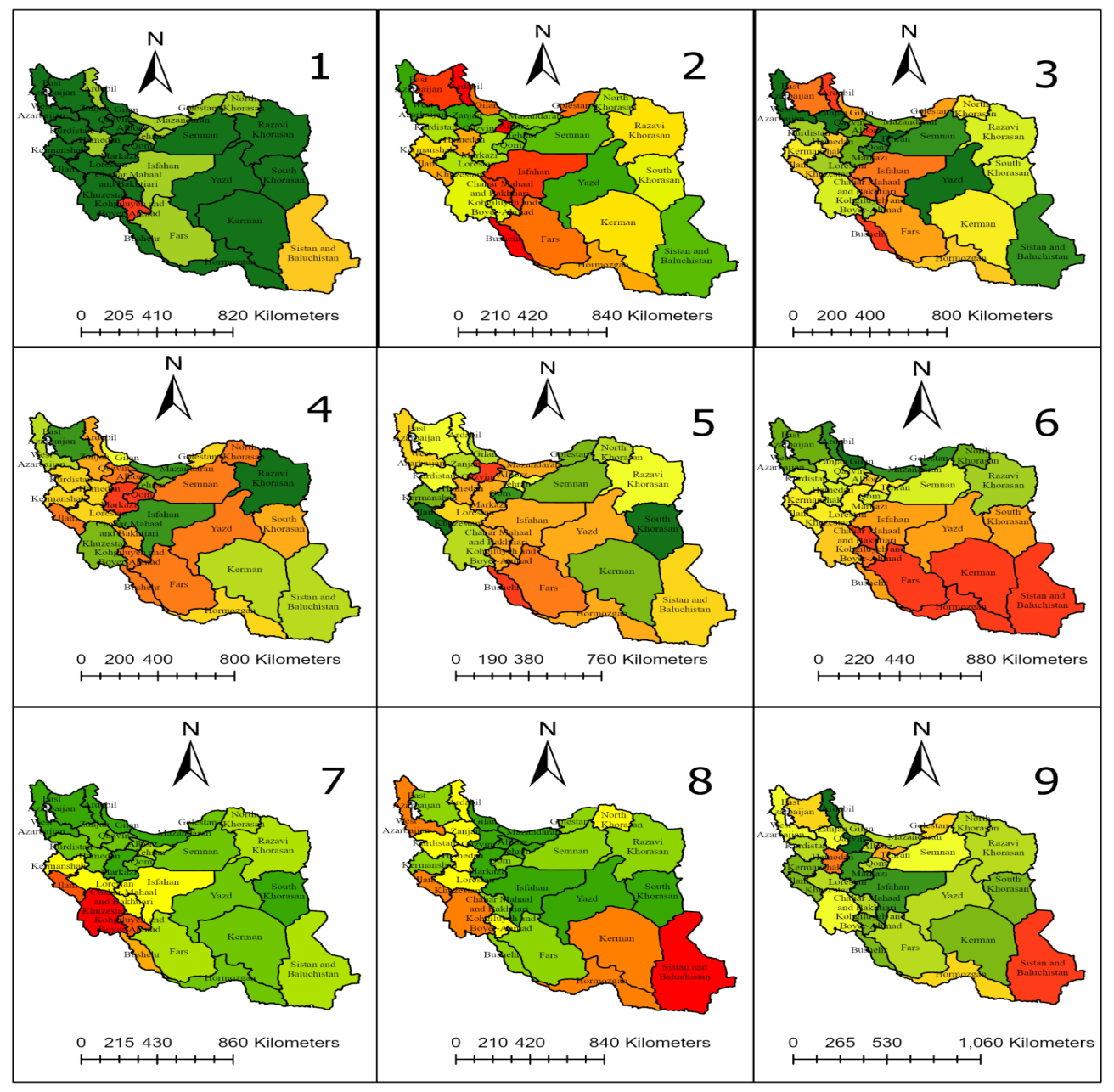


Fig.S3. Popularity analysis of the interaction of each explanatory variable on AAIR of neoplasms; (1) DALYs of STD, (2) DALYs of HBV, (3) DALYs of (HCV), (4) Number of healthcare centers, (5) Tobacco consumption rates (%), (6) UV index, (7) Annual mean concentration of PM 2.5, (8) Literacy rate, and (9) Gini coefficient (This map was generated using the tool of ArcGIS pro 2.5. (ESRI, Redlands, CA, USA, http://www.esri.com).
